# Specular Surface Shape Measurement with Orthogonal Dual-Frequency Fourier Transform Deflectometry

**DOI:** 10.3390/s23020674

**Published:** 2023-01-06

**Authors:** Zhiming Li, Dayi Yin, Yuanyu Yang, Quan Zhang, Huixing Gong

**Affiliations:** 1 Shanghai Institute of Technical Physics, Chinese Academy of Sciences, Shanghai 200083, China; 2 School of Information Science and Technology, ShanghaiTech University, Shanghai 201210, China; 3 School of Electronic, Electrical and Communication Engineering, University of Chinese Academy of Sciences, Beijing 100049, China

**Keywords:** orthogonal dual-frequency fringe, three-dimensional shape measurement, Fourier transform deflectometry, specular surface measurement

## Abstract

Three-dimensional (3D) shape measurement for specular surfaces is becoming increasingly important in various applications. A novel orthogonal dual-frequency fringe is proposed in the specular surface shape measurement to overcome the phase jumping and discontinuities in spatial phase unwrapping. The fringe recalibrated high-accuracy phase information from its high-frequency fringe component with low-ambiguity phase information from its low-frequency fringe component. An improved Fourier transform deflectometry method based on the orthogonal dual-frequency fringe is proposed to measure 3D specular surface shapes. Simulation results showed that the orthogonal dual-frequency Fourier transform deflectometry (ODD) method could precisely reconstruct flat surfaces with an error of 2.16 nm rms, and concave surfaces with an error of 1.86 μm rms. Experimental results showed that the reconstructed shapes of both the flat mirror and the concave mirror measured by the ODD measurement system were highly comparable to those obtained by the phase-measuring deflectometry (PMD) method. This new fringe provides a distinctive approach to structured pattern construction and reduces the phase unwrapping ambiguities in specular surface shape measurement. The ODD method can achieve accurate 3D shape measurement for specular surfaces by sampling only one fringe, providing a possible basis for future real-time measurement of specular surfaces.

## 1. Introduction

Specular surfaces such as reflective mirrors are widely utilized as crucial components in systems or applications in aerospace, precision instrumentation, the automotive industry, and the electronics industry [1,2,3,4]. Reflective mirrors are mainly used to reflect optical images, and their surface shape directly or indirectly influences the overall performance of a system. Therefore, there are usually precise requirements for the shapes of specular surfaces, and the measurement and reconstruction of specular surface shape is a crucial research field [2,5].

The three-dimensional (3D) measurement and reconstruction of specular objects differ from diffuse reflecting objects, since the specific light reflected by specular surfaces can only be observed through specific angles, and surface shapes cannot be directly observed. Three-dimensional reconstruction algorithms for general scenes or diffuse reflecting objects do not perform well in terms of specular objects. Therefore, the 3D reconstruction and measurement of specular objects is also of great interest. Currently, 3D specular surface shape measurement methods are divided into two main categories according to the measurement principle: interferometry [6,7] and deflectometry [5]. The interferometry method uses the difference between the reference path and the measurement path to calculate the 3D shape of the measured specular surface. Interferometry has an extremely high measurement accuracy and is widely used to measure the shape of high-precision mirrors. However, limited by the measurement wavelength of the measuring light, the measuring range of interferometry is relatively small, and interferometry cannot measure the 3D shape of rough specular surfaces and moving surfaces. The other kind of method is the deflectometry method [8,9,10,11,12,13,14]. Deflectometry methods use the reflection property of the specular surface to construct a quantifiable reflective optical path, determine the gradient of the mirrors, and reconstruct the surface shape [2]. The accuracy of deflectometry methods is in the same order as interferometry; however, deflectometry methods have a broader range of measurement than interferometry. In addition, they can also measure discontinuous specular surfaces [5]. The general steps of the deflectometry method include extracting phase information from the sampling fringe images deformed by the reflections of the tested surfaces [12] and unwrapping phase information to identify the corresponding camera pixels, surface points, and screen pixels [13]. The gradients of tested surfaces can then be calculated, and surface shapes can be reconstructed via the slope integration method [15,16,17]. Osten et al. [9] proposed that the surface shape of a specular object can be calculated from the change in an image after specular reflection, and they developed the phase-measuring deflectometry (PMD) to measure the specular surface shape. Peng Su et al. [14] proposed a software configurable optical test system (SCOTS) to measure the sizeable primary mirror of astronomical telescopes.

In order to obtain the phase distribution, several fringe patterns in two orthogonal directions are usually required to complete the measurement process in the phase-measuring method [18,19]. Composite patterns are designed to reduce the number of images and conduct the real-time measurement of 3D specular surface profiles [20]. Lei Huang et al. [12] proposed a monoscopic fringe reflectometric system with the assistance of the windowed Fourier ridges method, in which only one cross-fringe is used to reconstruct the changes in 3D surface shape. Another way to decrease the image number is to employ a color-encoded fringe pattern. Yuxiang Wu et al. [21] proposed a three-channel encoded fringe reflection technique to achieve a dynamic specular surface measurement. Trumper et al. [11] presented an instantaneous phase-shifting deflectometry method based on multiplexing phase-shifted fringe patterns with color, involving decomposition using the Fourier technique. However, compared with the structured light technique [22], fewer fringe patterns were proposed in the 3D shape measurement for specular surfaces. The fringe patterns for one-shot measurement are especially rare, which limits the development of the real-time measurement for specular surface. Furthermore, the decrease in image numbers simplifies the measurement process, but the phase jumping and discontinuities in the conventional spatial phase unwrapping reduce the stability when measuring complicated specular surfaces or discontinuous surfaces [18,23,24,25,26]. In the field of dynamic 3D shape measurement, two-frequency fringe projection profilometry employs an additional group of phase-shifting patterns with lower frequency to assistant pixel-by-pixel phase unwrapping, avoiding the phase ambiguities introduced by sinusoidal patterns and the arctangent function [18,24]. Research showed that another frequency pattern can assist phase unwrapping and lower the influence of phase ambiguity [18,24,25,26].

To improve the stability and the accuracy of phase unwrapping, a novel orthogonal dual-frequency fringe is proposed in the specular surface shape measurement to overcome the phase jumping and discontinuities in spatial phase unwrapping. The fringe recalibrated high-accuracy phase information from its high-frequency fringe component with low-ambiguity phase information from its low-frequency fringe component. To achieve a stable and efficient shape measurement of specular surfaces, an improved orthogonal dual-frequency Fourier transform deflectometry (ODD) method is presented in this paper. ODD can use the same system schematic with phase-measuring deflectometry and achieves the same accuracy while using only one image to extract phase information. An ODD fringe pattern simulation model is designed to simulate the fringe patterns deformed by different specular surfaces, and the performance of ODD is tested. An experiment based on a PMD measurement system was conducted to test the performance of the ODD system. The measurement experiment results also showed that the reconstructed shapes of both the flat mirror and the concave mirror measured by the ODD measurement system were highly comparable to those obtained by the phase-measuring deflectometry (PMD) method. This paper is organized in the following way: Section 2 explains the basic principles of the ODD method; Section 3 verifies the phase relationship between the frequency components of the fringe patterns and the results of the ODD simulation; Section 4 shows the experimental results of the ODD measurement system; Section 5 offers conclusions to this work.

## 2. Principles

### 2.1. The Principle of ODD

**Figure 1:**
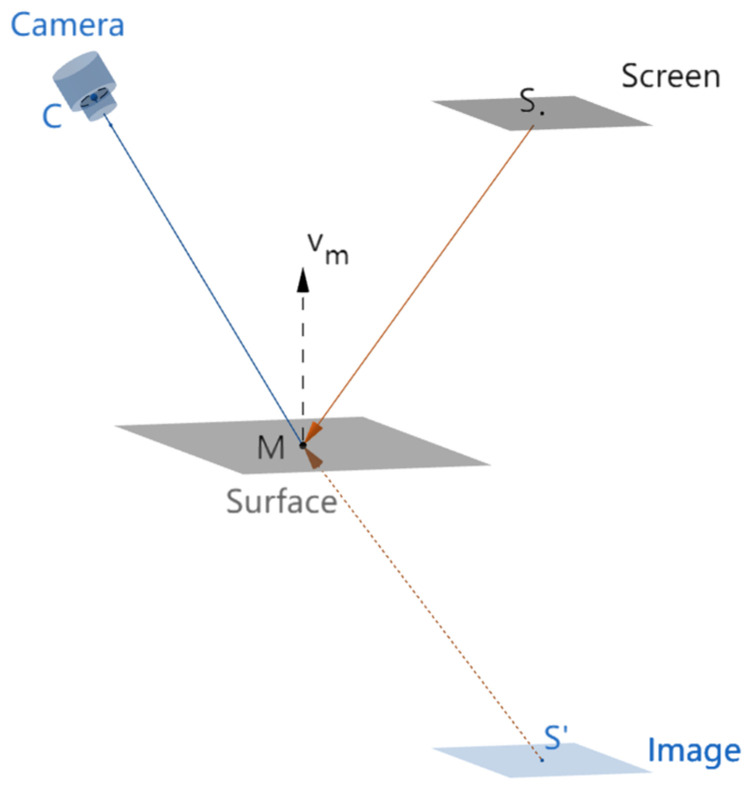
The schematic diagram of the deflectometry measurement system. Point M represents an arbitrary point on the surface under test. Point S represents the point corresponding to M on the screen, and point S’ represents the virtual image of S on the virtual image of the screen. Point C represents the point corresponding to M in the camera. Vector $v_{m}$ represents the normal vector of point M on the surface under test.

The deflectometry method applies vision techniques to detect the optical path and calculate surface slopes to reconstruct specular surface shapes. A basic measurement system comprises a hardware measurement platform and postprocessing algorithms. A general windowed discrete Fourier transform deflectometry measurement platform is demonstrated in Figure 1. It consists of a screen to display patterns, a high-speed camera with an imaging lens to capture the distorted patterns reflected by the specular surface under test, and a measurement plane as the reference plane. In order to measure the slope of the tested surface, the measurement system tracks reflection paths and builds geometric mapping among camera pixels, screen pixels, and specular surface points [9,12]. Generally, the measurement system displays a predesigned sinusoidal fringe pattern one or more times, and the high-speed camera captures the deformed sinusoidal fringe patterns reflected by the specular object under test. By preprocessing the image, including image distortion correction, extracting and unwrapping the phase information, and calculating normal vectors of specular surface points, the postprocessing algorithms can compute the slope distribution of the mirror surface under test and reconstruct the specular shape via integration methods [5,15,16,17].

Unlike most deflectometry methods, the ODD method focuses on the phase retrieval step and introduces the composite fringe pattern to improve the accuracy of phase information. The windowed discrete Fourier transform (WDFT) method [27,28,29] is employed to extract the phase information of the specific frequency fringe. WDFT firstly implements two-dimensional (2D) discrete Fourier transform on the deformed fringe pattern, extracts the specific frequency component from the deformed fringe pattern, and calculates the phase information involved in the frequency component. The basic principle of the dual-frequency Fourier transform method is similar to that of WDFT, and two specific frequency components are extracted. Its fringe pattern contains two sinusoidal fringe components in both orthogonal directions. In each direction, there are a low-frequency fringe component and high-frequency fringe component. The deformed fringe pattern of ODD can be expressed as

(1) $$I(x,y)=A+B\cos(\omega_{1}x+\varphi_{1})+B\cos(\omega_{2}x+\varphi_{2})+B\cos(\omega_{1}y+\varphi_{1})+B\cos(\omega_{2}y+\varphi_{2})$$

where *A* is the background intensity of fringe patterns, *B* is the magnitude intensity of sinusoidal components in the fringe pattern, $\omega_{1}$ and $\omega_{2}$ are the spatial frequencies of two sinusoidal fringe components, and when $\omega_{1}<\omega_{2}$, $\varphi_{1}$ and $\varphi_{2}$ are initial phases of the two sinusoidal fringe components.

**Figure 2:**
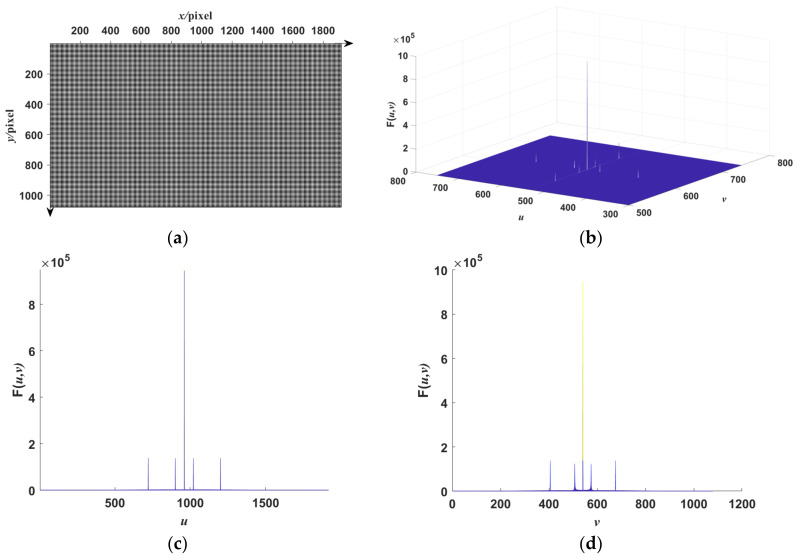
A simulation dual-frequency sinusoidal fringe and its frequency distribution in *x*- and *y*-directions. (**a**) Simulation dual-frequency sinusoidal fringe pattern generated by Matlab with $\omega_{1}=\pi /4$ rad/pixel and $\omega_{2}=\pi /16$ rad/pixel. Its resolution is 1920 × 1080 pixels. Note that the figure is a part of the fringe pattern. (**b**) The frequency spectrum of the fringe image. (**c**) The fringe’s frequency spectrum in the *u*-direction. (**d**) The fringe’s frequency spectrum in the *v*-direction.

The functions of the two frequency components are different. A typical orthogonal dual-frequency fringe pattern is shown in Figure 2 alongside its frequency spectrum generated by the fast Fourier transform method in the *x*- and *y*-directions. As is shown in Figure 2b, there are two frequency components in both directions, and the frequency components are set to guarantee that they are independent from each other. The high-frequency fringe component is utilized for phase retrieval and the further reconstruction of surface shape, and the low-frequency fringe component is introduced to assist the phase retrieval of the high-frequency fringe component.

The image is transformed to the frequency domain via the two-dimensional (2D) discrete Fourier transform [30] to extract the specific frequency components from the deformed fringe patterns captured by the camera. The process can be denoted as

(2) $$F(u,v)=\sum_{x=0}^{M-1}\sum_{y=0}^{N-1}f(x,y)e^{j2\pi (ux/M+vy/N)}$$

where f(x,y) is a digital image with a resolution M×N pixels, and F(u,v) is the result after 2D discrete Fourier transform. 

In the frequency domain, there are two frequency peaks in both directions in the orthogonal dual-frequency fringe patterns, as shown in Figure 2c,d. According to the windowed Fourier transform [27,28,29], for each direction, the first two maximums, except for the region around the background intensity, are extracted and sorted, and the effective frequency ranges around these two maximums are first extracted using a specific 2D bandpass filter. A threshold filter is used to filter out the frequency noise and retain the high amplitude of the extracted frequency domain. A windowed Gauss filter can be applied to reduce noise further and extract the effective frequency range if needed. Inverse discrete Fourier transform is then applied to the extracted frequency domain to recover the phase information. This process can be denoted as

(3) $$g(x,y)=F^{-1}[H(u,v)B(u,v)F(u,v)]$$

where $F^{-1}$ is the inverse discrete windowed Fourier transform (IDFT) process, B(u,v) is a specific bandpass filter, H(u,v) is a Hanning window as the response of the band pass filter [28], and g(x,y) is the result of this process. The bandpass filter is utilized for acquiring a specific frequency band. For specific frequency components, its center is set at the frequency of the designed frequency value, and its frequency range should make sure there is no spectral aliasing in the extracted spectrum. As we obtain g(x,y), the wrapped phase map in the preset frequency domain can be obtained according to the windowed Fourier transform [27,28,29] by

(4) φ(x,y)=arctanIm[g(x,y)]Re[g(x,y)]

where Im[g(x,y)] is the imaginary part, and Re[g(x,y)] is the real part of g(x,y). φ(x,y) is the phase map of the extracted frequency component in the deformed fringe pattern. It is important to note that the inverse tangential function is the 2D inverse tangential function and all the phase maps extracted from the camera image are not continuous, which will introduce phase truncation when the actual phase absolute value is larger than 2π. The wrapped phase map therefore needs to be unwrapped to establish the mapping between camera pixels and screen pixels. For the phase unwrapping algorithm, this paper introduces the quality map guided phase unwrapping algorithm [31,32] as the primary phase unwrapping process to obtain the continuous phase map.

Once the continuous phase distribution is obtained, the absolute phase map should be recalibrated, referring to the reference point. Since the relative phase difference between measured phase points and the reference point is constant, the recalibration of the continuous phase can be expressed as

(5) $$\varphi_{abs}=\varphi_{f}^{}-\varphi_{m}^{}+\varphi_{ref}^{}$$

where $\varphi_{f}$ is the measured phase map, $\varphi_{m}$ is the measured phase value of the reference point, and $\varphi_{ref}$ is the reference phase value of the reference point. The reference phase of the reference point is obtained by lighting a specific point on the screen and finding it on the image during the measuring process. As known from Equation (1), the absolute phase maps can be converted to screen pixel coordinate maps via a transformation with the following relationship:

(6) $$\left[\begin{array}{c} x_{v}\\ y_{v}\\ 1 \end{array}\right]=\left[\begin{array}{ccc} 2\pi /\omega_{x} & 0 & 0\\ 0 & 2\pi /\omega_{y} & 0\\ 0 & 0 & 1 \end{array}\right]\left[\begin{array}{c} \varphi_{x}\\ \varphi_{y}\\ 1 \end{array}\right]$$

where $\omega_{x}$ and $\omega_{y}$ are the spatial frequency of sinusoidal fringes in the *x*- and *y*-directions, and $\varphi_{x}$, $\varphi_{y}$ are the absolute phase maps in the *x*- and *y*-directions.

For the current orthogonal dual-frequency fringe pattern, we can obtain the continuous phase map in each of the four frequency bands via the above process. In each direction, there are two phase maps extracted and retrieved, and they are not independent of each other. There is a linear relationship between two phase maps in the same direction, which provides the theoretical basis for the ODD measurement system.

### 2.2. The Phase Relationship between Two Frequency Components in the Same Direction

Note that the preset sinusoidal fringe pattern contains two fringe components in the horizontal and vertical directions. In each direction there are two sinusoidal fringe components with different frequencies. In the phase-measuring deflectometry method [2,8,11], the horizontal fringes are used to determine the vertical y-coordinate in screen coordinate system after phase calculation and phase unwrapping. Similarly, the vertical fringes can determine the horizontal *x*-coordinate in the screen coordinate system. The screen coordinate system’s origin is in the upper left corner, and its *x*- and *y*-axes are parallel to the two screen edges, correspondingly. For ODD, the fringe components in different directions determine the coordinate in different directions and establish a unique coordinate in the screen coordinate system. This process can be accessed through

(7) $$\left\{\begin{array}{c} x=(\varphi_{\omega_{x}}^{x}-\varphi_{abs}^{x}+\varphi_{ref}^{x})/\omega_{x}\\ y=(\varphi_{\omega_{x}}^{y}-\varphi_{abs}^{y}+\varphi_{ref}^{y})/\omega_{y} \end{array}\right.$$

where x and y are the corresponding screen pixel coordinate of camera pixels, $\varphi_{\omega_{x}}^{x}$ and $\varphi_{\omega_{y}}^{y}$ are the phase maps in the *x-* and *y*-directions under frequency $\omega_{x}$ and $\omega_{y}$ calculated by IDFT, and ($\varphi_{abs}^{x},\varphi_{abs}^{y}$) is the measured phase coordinate of the reference point. ($\varphi_{ref}^{x}$,$\varphi_{ref}^{y}$) are the reference phase values of the reference point. For the two sinusoidal fringes in both directions, we can obtain two sets of corresponding screen pixel coordinates by following the above method, and the two sets of corresponding screen pixel coordinates of the same points are equal because they correspond to the same point on the screen. Thus, we can obtain the transformation between the low-frequency fringes and the high-frequency fringes as follows:

(8) $$\varphi_{\omega_{2}}=\frac{\omega_{2}}{\omega_{1}}\varphi_{\omega_{1}}+\frac{\omega_{2}}{\omega_{1}}(\varphi_{ref1}-\varphi_{abs1})+\varphi_{abs2}-\varphi_{ref2}$$

where $\varphi_{\omega_{1}}$ and $\varphi_{\omega_{2}}$ are the phase maps under low and high frequencies, correspondingly. $\omega_{1}$ is low frequency and $\omega_{2}$ is high frequency. $\varphi_{ref1}$ and $\varphi_{ref2}$ are the reference phases of the reference point. $\varphi_{abs1}$ and $\varphi_{abs2}$ are the absolute phases of the reference point. Note that the reference points under different frequencies are the same, so their reference phases can also be transformed from each other. The parameters in Equation (8) are all constant except $\varphi_{\omega_{1}}$ and $\varphi_{\omega_{2}}$, so there is a linear relationship between $\varphi_{\omega_{1}}$ and $\varphi_{\omega_{2}}$, which can be simplified as

(9) $$\varphi_{\omega_{2}}=\frac{\omega_{2}}{\omega_{1}}\varphi_{\omega_{1}}+C$$

where $C=\frac{\omega_{2}}{\omega_{1}}(\varphi_{ref1}-\varphi_{abs1})+\varphi_{abs2}-\varphi_{ref2}$.

The measurement accuracy of the deflectometry measurement systems is influenced by the frequency of sinusoidal fringe patterns [33]. Compared with the high-frequency sinusoidal fringe, the low-frequency fringe has more sampling points in one period on the same screen, and its phase difference between adjacent points is relatively small. When influenced by noise or measuring large-variation surface, the low-frequency fringe is less likely to cause a major phase change of more than 2π compared with the high-frequency fringe, which makes phase unwrapping easier but lowers the measurement accuracy. In contrast, the high-frequency fringe is more sensitive to surface shape changes due to its high phase difference among adjacent points, and a higher accuracy measurement within a single period can be obtained. However, when the adjacent phase change is larger than 2*π*, this will cause a phase loss in the wrapped phase map, introducing errors in surface shape measurement. The combination of two fringes can achieve a higher and more stable measurement.

**Figure 3:**
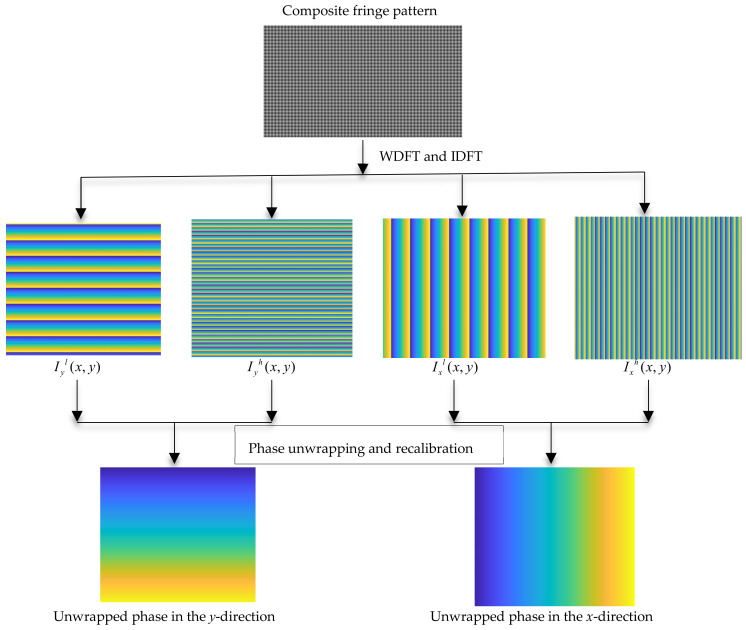
The overview of phase extraction, unwrapping, and recalibration process in ODD. $I_{y}^{l}(x,y)$ and $I_{y}^{h}(x,y)$ are the wrapped phase maps of the low- and high-frequency fringe components in the *y*-direction, and $I_{x}^{l}(x,y)$ and $I_{x}^{h}(x,y)$ are the wrapped phase maps in the *x*-direction. Clear phase maps are shown in the following figures.

In ODD, fringes with different frequencies in the same direction provide two distinct phases. It is evident in Equation (1) that the gray scale of the fringes varies for different screen pixels. It can be found from Equation (9) that the phase values of the same screen pixel under different frequencies can be obtained if one of them is known. Therefore, the phase map of the low-frequency fringe can be utilized to assist in the recalibration in the phase map of the high-frequency fringe. The first step of building the phase map of high-frequency fringe is to perform WDFT and IDFT to obtain the wrapped phase map and then apply the phase unwrapping algorithm to obtain the continuous phase map. A reference high-frequency phase map can be obtained from Equation (9) and is then used to check and correct the range of high-frequency fringes. This recalibrates the whole phase map and improves the accuracy of the WDFT deflectometry measurement. However, in practice, due to the constraints of the calibration conditions and the difference in measurement sensitivity between the two fringes, the calibration process may not run as the equation described; as the fast Fourier transform process is discrete, $\omega_{2}/\omega_{1}$ is not strictly equal to the theoretical value, and an interval is established to help the recalibration run smoothly. In this paper, we relax the constraint to ±π scale. This phase extraction, unwrapping, and recalibration process is shown in Figure 3.

Please note that the radial and tangential distortion of the captured fringe was corrected in the image processing step according to the camera calibration method [34]. Under the condition that the measurement system is precalibrated, and the phase map is determined, the corresponding physical coordinates of each camera pixel can be obtained after the camera calibration [34], which can be expressed by

(10) $$s{\left[\begin{array}{c} u_{v}\\ v_{v}\\ 1 \end{array}\right]}^{T}=ins_{v}\cdot \left[\begin{array}{c} x_{v}\\ y_{v}\\ z_{v} \end{array}\right]$$

where *s* is an arbitrary scale factor, and $ins_{v}=\left[\begin{array}{ccc} 1/f_{x} & \gamma & O_{x}\\ 0 & 1/f_{y} & O_{y}\\ 0 & 0 & 1 \end{array}\right]$ is a transformation associated with the screen intrinsic parameters. This converts the view coordinate into the view pixel coordinate. Factors $f_{x}$ and $f_{y}$ are the width and the height of the screen pixel, respectively, $\left(O_{x},O_{y}\right)$ is the physical coordinate of the view coordinate system’s origin [35], and the parameter γ describes the skew of the two image axes.

Therefore, the maps among every camera coordinate C:$\left(x_{c},y_{c},z_{c}\right)$, screen coordinate S:$\left(x_{s},y_{s},z_{s}\right)$, and the corresponding reference mirror point M:$\left(x_{m},y_{m},z_{m}\right)$ are built, and then the direction of incident light ray and the reflected light ray at mirror points can be determined. The unit vector $\vec{i}$ of the incident light and the unit vector $\vec{r}$ of the reflected light at the mirror point can then be determined. The normal vector $\vec{n}$ of each mirror point can be calculated [10]. This process can be expressed by

(11) $$\vec{n}=-\vec{i}+\vec{r}$$

The slope of the mirror point can then be obtained from the normal vector $\vec{n}$, and the 3D shape of the mirror can be reconstructed by the zonal integration method. 

## 3. Simulation

A simulation was conducted to verify the phase relationship between low- and high-frequency fringe components in orthogonal dual-frequency fringe patterns. In addition, a simulated deformed fringe pattern based on the real measurement system and the ideal shape of the specular surface was generated via a simulated pattern generation model [35]. This fringe image was introduced to simulate the measuring process and test the performance of the ODD method.

The system configuration in this experiment is based on a typical PMD measurement system. It consists of an LCD screen with a resolution of 1920 × 1080 pixels, a high-speed camera with a resolution of 2048 × 2048 pixels at a frame rate of 30 frames per second (FPS) with a 16 mm lens, and a regulable flat stage. The fringe pattern displayed on the screen was generated by codes, and the camera was calibrated using Zhang’s camera calibration method [34]. Before conducting the simulation and the test, the measurement platform completed the establishments of coordinate systems, the geometric calibration, and the camera calibration following the conventional PMD calibration method [36]. Camera calibration and geometric calibration in our paper were performed in the meantime. The lens was first set to focus on the middle point between the mirror surface and the virtual image of the screen so that the camera could clearly capture the checkerboard both on the test stage and the screen. Note that the same size checkerboard was placed on the test stage, and the influence of the height of the checkerboard was eliminated by adjusting the height of the test stage. A precise mirror was also placed on the test stage so that the camera could capture the virtual images of the checkerboard displayed on the screen and the checkerboard on the test stage in one image. We adjusted the focal length and the aperture of the lens to make sure both checkerboards were clear in the image, and fixed the camera aperture and the focal length. In the following step, we removed the standard mirror, and different views of the checkerboard were acquired when the pose of the checkerboard changed. A special pose was set and recorded to calibrate the geometric relationship between the test stage and the camera. After that, a standard mirror was then placed on the test stage to reflect the same checkerboard displayed on the screen, and the camera captured the views to calibrate the geometric relationship between the screen and the camera. The captured views of the checkerboard were used to calibrate the camera via Zhang’s method. Through the above process, the geometric relationship and the camera parameters were calibrated.

### 3.1. Verification of the Orthogonal Dual-Frequency Fringe Relationship under Ideal Conditions

**Figure 4:**
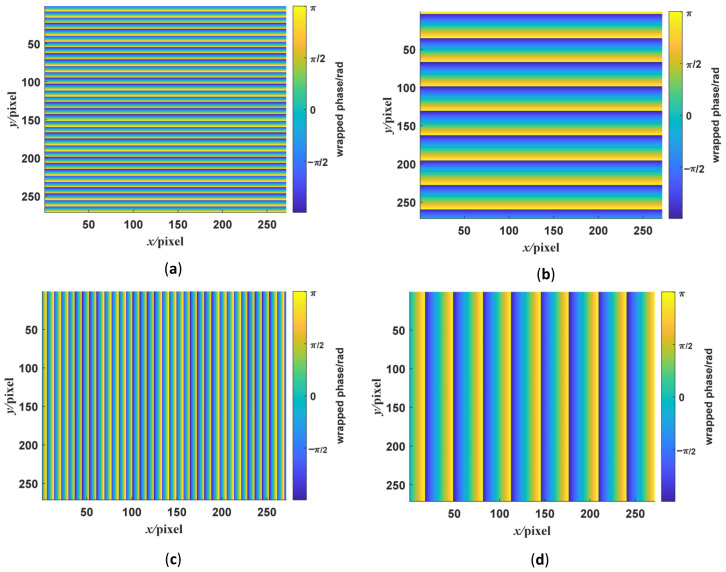
The wrapped phase maps of the displayed dual-frequency sinusoidal fringe calculated by WFT. The phase maps shown in the figures are the part of the whole phase maps. Panels (**a**,**b**) are the phase maps of the high- and low-frequency fringes in the *y*-direction. Panels (**c**,**d**) are the phase maps of the high- and low-frequency fringes in the *x*-direction.

**Figure 5:**
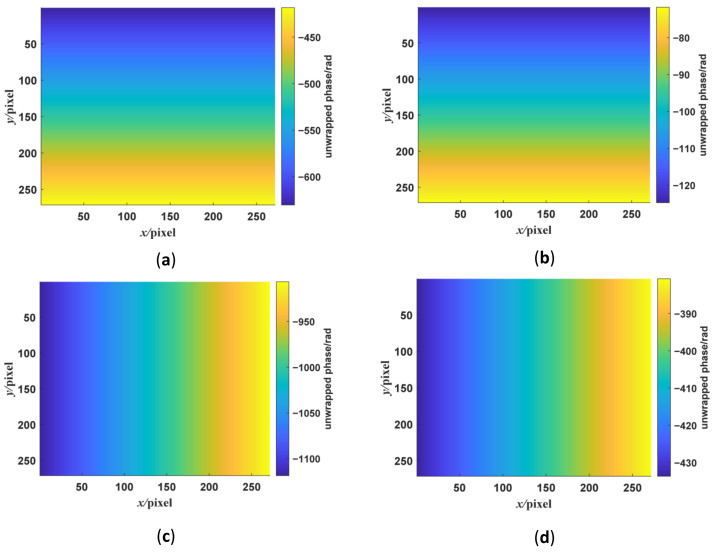
The phase maps of displayed dual-frequency sinusoidal fringe unwrapped by quality-guided phase unwrapping algorithm. (**a**,**b**) The unwrapped phase maps of high- and low-frequency fringes in the *y*-direction. (**c**,**d**) The unwrapped phase maps of high- and low-frequency fringes in the *x*-direction.

**Figure 6:**
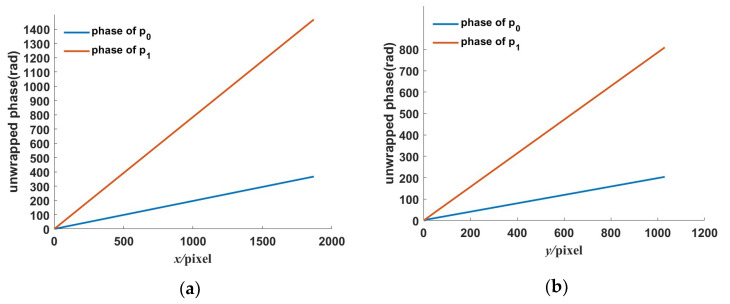
The phase relationship between two different frequency fringes in the *x*-direction and *y*-direction. The phase maps are from the same line in the deformed fringe pattern. $p_{0}$ is the unwrapped phase of the low-frequency fringe component and $p_{1}$ is the unwrapped phase of the high-frequency fringe component. (**a**) The *x*-coordinate phase relationship. (**b**) The *y*-coordinate phase relationship.

For the fringe pattern displayed on the screen, the composite fringe pattern can be expressed by

(12) $$I(x,y)=0.25\cos(\frac{\pi }{4}x)+0.25\cos(\frac{\pi }{16}x)+0.25\cos(\frac{\pi }{4}y)+0.25\cos(\frac{\pi }{16}y)$$

where we set the frequencies in two directions with the low-frequency fringe at π/16 rad/pixel, the high-frequency fringe at π/4 rad/pixel, and the background gray scale at 0. The standard fringe pattern image is shown in Figure 2a. Its frequency spectrum is shown in Figure 2b. There are two frequency components in the frequency spectrum. We extract the low- and high-frequency component by using WDFT, and the extraction window size is set to 30. After WDFT and IDFT, the wrapped phase map is shown in Figure 4. Figure 4a,b show the wrapped phase maps of the high- and low-frequency fringes in the horizontal direction, respectively, and Figure 4c,d show the wrapped phase maps of the high- and low-frequency fringes in the vertical direction. Figure 4 indicates that the WDFT process can extract the wrapped phase information of the specific frequency component from the orthogonal sinusoidal fringe pattern. The continuous phase map of the high-frequency fringe after phase unwrapping and recalibration is shown in Figure 5. To better show the linear relationship, the phase maps of the same line with different frequency components were taken out and placed on the same start point. The linear relationship between two phase maps is shown in Figure 6. It can be seen from Figure 6a that, for the continuous phase map of pixels of the same column in different frequency, the phase map in Figure 6 is recalibrated to place them on the same phase start point.

### 3.2. The Performance of Recalibrated Phase Unwrapping

**Figure 7:**
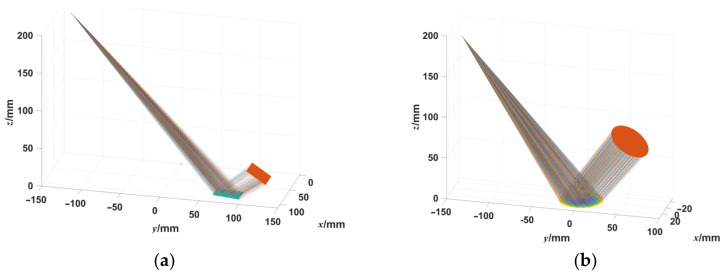
The ODD fringe simulation models. (**a**) The fringe simulation model based on the ideal flat mirror (only display a part of the flat mirror). (**b**) The fringe simulation model based on the ideal concave mirror.

In order to examine the ideal performance of the ODD measurement system, based on the PMD measurement system simulation model [32], a fringe simulation model based on the ODD measurement system was constructed, as shown in Figure 7. The model was based on the configuration of the actual measurement system, retraces the optical path of specular surface measurement, and generates the orthogonal dual-frequency sinusoidal deformed fringes with different surface shapes.

**Figure 8:**
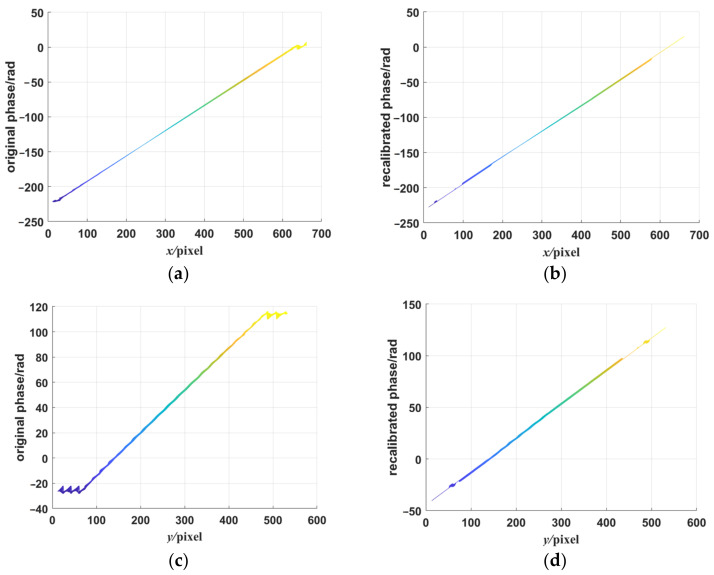
The unwrapped phase maps from the high-frequency fringe components and their recalibrated phase maps by the corresponding low-frequency fringe components. (**a**,**c**) The unwrapped phase maps from the high-frequency fringes in the *x*- and *y*-directions. (**b**,**d**) The unwrapped phase maps recalibrated by the corresponding low-frequency fringes in the *x*- and y-directions.

To compare the performances between the single-frequency fringe pattern and the orthogonal dual-frequency fringe pattern in the phase unwrapping process, we simulated a spherical surface shape as the tested plane and generated the deformed fringe via the fringe simulation model. Unwrapped phase maps extracted from high-frequency fringe components in both directions and corresponding recalibrated phase maps are shown in Figure 8. As shown in Figure 8a,c, there were wrapped phases in the bottom and the top of the phase map. These wrapped phases may be caused by some reasons such as the large shape variation in the unwrapped direction and the limitations of the spatial phase unwrapping algorithm. The recalibration step could effectively correct the errors of the unwrapped phase according to the results shown in Figure 8b,d. The results showed that the combination of the low-frequency fringe component and the high-frequency fringe component could improve the phase jump problem of single-frequency fringe patterns. However, there were some phase jumps that remained in the recalibrated phase maps, and we hope we can improve the problems in our further study.

### 3.3. The Measurement Results in Simulation Shape Measurement

**Figure 9:**
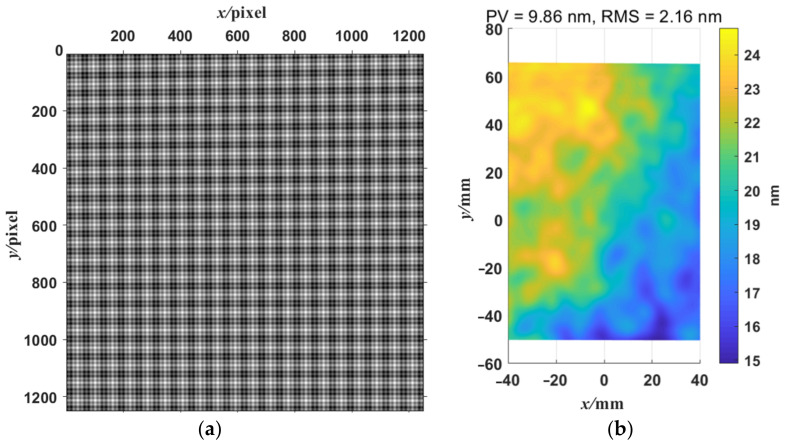
The simulated fringe pattern deformed by the flat mirror and its 3D shape reconstructed by ODD. (**a**) The simulated fringe patterns of the ODD fringe simulation model. (**b**) The reconstructed shape of the flat mirror by ODD.

**Figure 10:**
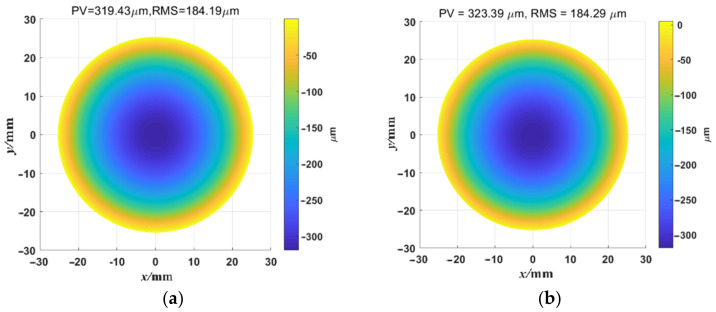

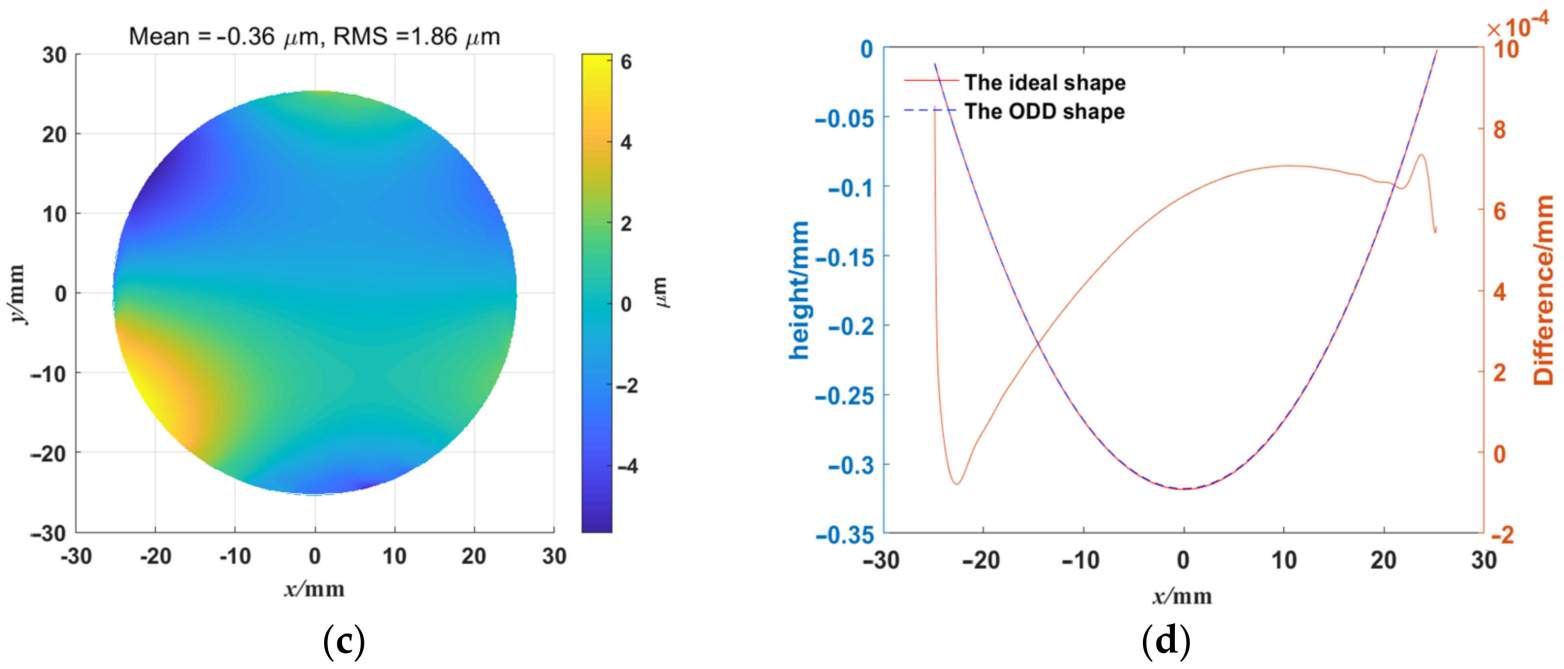
Three-dimensional shape of the simulated measured concave mirror and the reconstructed concave mirror, and the difference between the ideal shape and the reconstructed shape. (**a**) The ideal shape of the concave spherical surface simulated by the fringe simulation model. (**b**) The 3D shape of the concave spherical surface reconstructed by ODD. (**c**) The error map of the ODD method when reconstructing the concave spherical surface. (**d**) The shape of the *y*-coordinate center cross-section and the difference between the two shapes.

For the ideal flat simulated mirror, as shown in Figure 7a, the simulation of the reflective surface is the same as the test stage, and the height of the surface is 0 mm. For the ideal simulated concave surface, a 50.8 mm diameter concave spherical surface with R = 1010 mm is simulated, and its fringe simulation model is built as shown in Figure 7b. The fringe pattern generated by the fringe simulation model is shown in Figure 9a. For an ideal flat specular surface, the shape of the mirror surface reconstructed by ODD is shown in Figure 9b. As demonstrated in Figure 9b, ODD can precisely reconstruct the surface shape of the ideal simulated flat mirror. The peak-to-valley (PV) value of the reconstructed shape is 9.86 nm, and its root mean square (RMS) value is 2.16 nm. As the height of the ideal simulated mirror is 0 mm, the PV value and the RMS value also showed the deviation of the ODD method in reconstructing flat mirrors under ideal conditions. The PV value and the RMS value show that ODD has excellent performance in reconstructing flat specular surfaces. For ideal simulated concave mirrors, the reconstructed results of the deformed fringe pattern based on the simulated spherical mirror (Figure 10a) are shown in Figure 10b. ODD can reconstruct the spherical surface shape to a level highly comparable to the simulated surface shape. Their difference map is shown in Figure 10c. It can be seen that ODD still has precise shape reconstruction performance for ideal simulated concave mirrors with relatively large changes in surface shape, and its accuracy is inferior to that of the flat mirror surface shape measurement, but it still achieves micron-level accuracy. The mean value of the difference is −0.36 μm, and the RMS value is 1.86 μm. Figure 10d shows the ODD performance for the reconstruction of concave shapes. The reconstructed shape cross-section is highly comparable to that of the ideal shape, and the difference between the two cross-sections is below 1 μm. The simulation data show that the dual-frequency WDFT can accurately measure the surface shape of flat mirrors as well as continuous mirrors with significant surface shape variations under ideal conditions.

However, it is noted that the error of ideal simulated concave mirrors reconstruction was higher than that of the ideal simulated flat mirror shape. There are some possible reasons for this. Firstly, we noted that high error regions were distributed on the edge of the concave mirror, while the errors of the reconstructed flat mirror shape were homogeneous. This may have been caused by the height variation in the reflection characteristics of the concave mirrors. For the deflectometric method, when the height between the mirror shape and the reference plane increased, the difference increased. The edges of the concave mirrors were relatively high compared with the center, so there may have been high edges. Another possible reason is that the rotation matrix and the translation vector theoretically calculated from the camera calibration were applied to the shape measurement of the near-reference shape. When the height was large, the matrix and the vector may be not applicable to the edge of the shape, resulting in an increased difference. In addition, phase errors in the phase unwrapping step may cause the difference. The phase unwrapping accuracy in the concave mirror shape reconstruction is lower than that in the flat mirror shape reconstruction; even after the recalibration step, there were small phase jumps in the phase maps, which are shown in Figure 8b,d. Further studies and experiments should be completed in the future to analyze the error source of the ODD method.

## 4. Experiment

**Figure 11:**
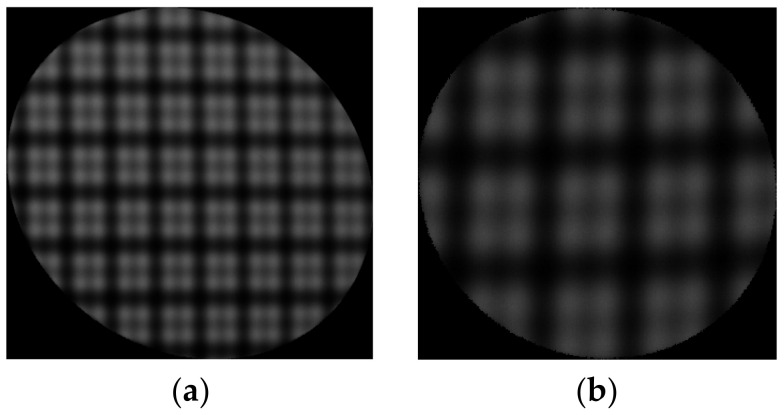
The orthogonal dual-frequency fringe patterns from the ODD measurement system. (**a**) The orthogonal dual-frequency fringe pattern deformed by the flat mirror. (**b**) The orthogonal dual-frequency fringe pattern deformed by the concave mirror.

**Figure 12:**
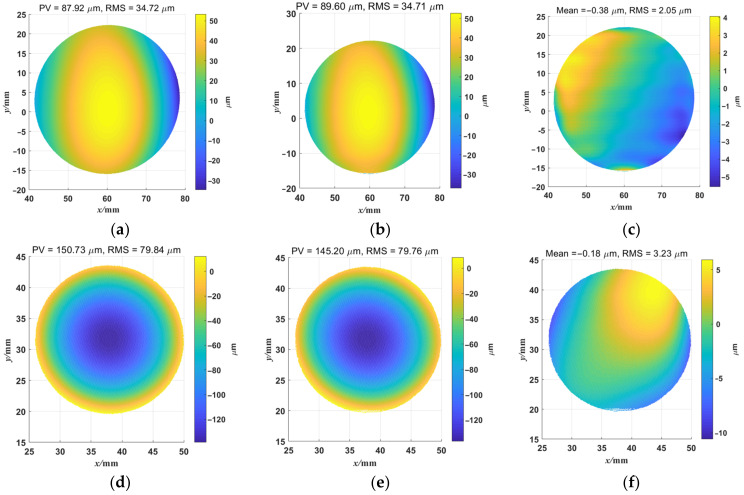
The measurement results of the ODD measurement system and the PMD measurement system. (**a**) The flat mirror shape reconstructed by PMD. (**b**) The flat mirror shape reconstructed by ODD. (**c**) The reconstructed flat shape difference between PMD and ODD. (**d**) The concave mirror shape reconstructed by PMD. (**e**) The concave mirror shape reconstructed by ODD. (**f**) The reconstructed concave shape difference between PMD and ODD.

In order to test the performance of the ODD measurement system, we introduced a PMD measurement system as the hardware platform of the ODD measurement system. Note that the system configuration and parameters of the ODD measurement system were identical to the simulation. Both flat and concave mirrors were tested. As the only significant difference between PMD and ODD measurement system was the displayed fringe patterns, and PMD methods are well-known methods in specular surface shape measurement [8,9,10,11], the PMD method was conducted as a reference to better contrast the measurement accuracy of the systems. For the ODD measurement system, we used an orthogonal dual-frequency sinusoidal fringe with a low frequency component of π/16 rad/pixel and a high-frequency component of π/8 rad/pixel for the surface shape measurement. There are 16 screen pixels involved in one high-frequency component and 32 screen pixels in one low-frequency component. The reason why the two frequency pairs were established is that lower-frequency components were difficult to extract from the frequency domain and they were too close to the constant component in the frequency domain in our system. Compared with the simulation, the frequency of the high-frequency fringe component decreased to π/8 rad/pixel due to the clarity of the captured deformed fringe patterns, and the low-frequency fringe component was unchanged. Please note that this change might decrease the accuracy of the shape reconstruction. However, the hardware platform and algorithms in the ODD measurement system are the same as the simulation, and the effectiveness of the ODD method was not changed. Fast Fourier transform and inverse fast Fourier transform were introduced to implement WDFT and IDFT; the shield factor was defined to guarantee the correct selected frequency and the quality-guided phase unwrapping algorithm; and the phase recalibration and the zonal integration method were utilized for the 3D shape reconstruction. For the PMD measurement system, the three-step phase-shifting method was used as the phase calculation method, and the same algorithms were used for phase unwrapping and integral reconstruction. The deformed fringe patterns from the ODD measurement system are shown in Figure 11. Note that there is distortion in Figure 11a; the orthogonal dual-frequency fringe pattern was deformed by the flat mirror, and was undistorted by the undistort function in the image preprocess step. Figure 11b shows the deformed fringe pattern from the concave mirror measurement. The reconstructed shapes from PMD and ODD are shown in Figure 12a,b, respectively. Their difference map is shown in Figure 12c. The results show that the ODD measurement system can reconstruct the overall shape of the flat mirror, and the shape difference between PMD and ODD was small. The PV and RMS values were very close. The overall mean value of the difference map was −0.38 μm, and its rms value was 2.05 μm. The results of reconstruction of the ideal simulated concave mirror are shown in Figure 12d,e. Both methods could accomplish the reconstruction of the concave mirror shape; the mean value of the difference map was −180 nm, and the RMS value was 3.23 μm. The differences between the two PV values of the two methods is noticeable, and the differences between the two reconstructed shapes are mainly distributed on the edge of the mirror. This is because the ODD method employed discrete Fourier transform to extract the phase information, and made large errors around the concave mirror edges. Overall, ODD achieves similar results to the PMD system, with differences in PV and RMS values on the micron scale. The micron differences might be improved by further tuning the measurement system, e.g., setting bias compensation, improving the calibration accuracy of the system, etc.

With similar measurement accuracy, ODD can achieve similar measurement accuracy to PMD while using only one sampled image, and the system components and architecture of the measurement system are identical. ODD provides a basis and opens new pathways for such methods to achieve dynamic surface shape measurement on specular surfaces.

## 5. Conclusions

This study presented a new deflectometry method based on the orthogonal dual-frequency fringe pattern and the windowed Fourier transform method to reconstruct the 3D shape of specular surfaces. The linear relationship between the phase maps of the low- and high-frequency components was verified. The recalibration from the orthogonal dual-frequency fringe effectively reduced phase unwrapping ambiguities in high-frequency phase unwrapping. The simulation results showed that ODD could reconstruct the flat mirror shape at a nanometer scale, and the concave mirror shape reconstruction with ODD was at the micron scale. The experimental results showed that the ODD method achieved the same accuracy in measuring flat and concave specular surfaces compared with the phase-measuring deflectometry method. Both the simulation model and experimental results demonstrated the viability of the proposed method. However, it has certain limitations in terms of sources of errors in the simulation for the concave mirror shape measurement. Further work needs to be carried out to estimate the robustness of the ODD method, and continued efforts are needed to make the ODD method more accessible to the real-time shape measurement for specular surfaces.

## Data Availability

Not applicable.
